# An integrated characterization of contractile, electrophysiological, and structural cardiotoxicity of *Sophora tonkinensis* Gapnep. in human pluripotent stem cell-derived cardiomyocytes

**DOI:** 10.1186/s13287-018-1126-4

**Published:** 2019-01-11

**Authors:** Ruiying Wang, Min Wang, Shan Wang, Ke Yang, Ping Zhou, Xueheng Xie, Qi Cheng, Jingxue Ye, Guibo Sun, Xiaobo Sun

**Affiliations:** 1 Key Laboratory of Bioactive Substances and Resources Utilization of Chinese Herbal Medicine, Ministry of Education, Institute of Medicinal Plant Development, Chinese Academy of Medical Sciences & Peking Union Medical College, Beijing, 100193 China; 2 Collaborative Innovation Center of Yangtze River Delta Region Green Pharmaceuticals, Zhejiang University of Technology, No.18, Chaowang Road, Xiacheng District, Hangzhou, 310014 Zhejiang China; 3 Harbin University of Commerce, Harbin, 150028 Heilongjiang China; 4 Beijing Health Olight technology Co., Ltd, Beijing, 100068 China

**Keywords:** Cardiotoxicity, *Sophora tonkinensis*, hiPSC-CMs, Cardio-NLCS

## Abstract

**Background:**

Cardiotoxicity remains an important concern in drug discovery and clinical medication. Meanwhile, *Sophora tonkinensis* Gapnep. (*S. tonkinensis*) held great value in the clinical application of traditional Chinese medicine, but cardiotoxic effects were reported, with matrine, oxymatrine, cytisine, and sophocarpine being the primary toxic components.

**Methods:**

In this study, impedance and extracellular field potential (EFP) of human-induced pluripotent stem cell-derived cardiomyocytes (hiPSC-CMs) were recorded using the cardio non-labeled cell function analysis and culture system (Cardio-NLCS). The effects of matrine, oxymatrine, cytisine, and sophocarpine (2, 10, 50 μM) on cell viability; level of lactate dehydrogenase (LDH), creatine kinase MB isoenzyme (CK-MB), and cardiac troponin I (CTn-I); antioxidant activities; production of reactive oxygen species (ROS) and malondialdehyde (MDA); and disruption of intracellular calcium homeostasis were also added into the integrated assessment.

**Results:**

The results showed that matrine and sophocarpine dose-dependently affected both impedance and EFP, while oxymatrine and cytisine altered impedance significantly. Our study also indicated that cardiotoxicity of matrine, oxymatrine, cytisine, and sophocarpine was related to the disruption of calcium homeostasis and oxidative stress. Four alkaloids of *S. tonkinensis* showed significant cardiotoxicity with dose dependence and structural cardiotoxicity synchronized with functional changes of cardiomyocytes.

**Conclusions:**

This finding may provide guidance for clinical meditation management. Furthermore, this study introduced an efficient and reliable approach, which offers alternative options for evaluating the cardiotoxicity of the listed drugs and novel drug candidates.

**Electronic supplementary material:**

The online version of this article (10.1186/s13287-018-1126-4) contains supplementary material, which is available to authorized users.

## Background

Cardiotoxicity is still one of the primary factors restricting the development of drugs. Drug development is a time-consuming and expensive process, and past statistics show that up to 90% of compounds that passed pre-clinical screening were terminated in a clinical trial, with approximately 45% of those failures being due to cardiotoxicity [1]. In addition, many drugs that were originally on the market have been withdrawn due to clinical cases of cardiotoxicity. The HERG channel in Chinese hamster ovary or human embryonic kidney cells has been found to be closely related to QT prolongation and torsade de pointes (TdP) and has become an important component of early drug screening and safety pharmacology experiments [2, 3]. However, the assessment of cardiotoxicity cannot be completely dependent on these simple model cell lines, as they do not reproduce the integrated cardiomyocyte system and had species differences [4]. For example, there were cases in which compounds that interacted with hERG may not have caused TdP, e.g., verapamil [3]. Besides, cardiac safety was evaluated by some complementary assays such as multi-electrode arrays (MEA) and impedance, which the current needs were still not meet [2]. Therefore, there is an urgent need for a method to comprehensively evaluate cardiotoxicity in both pre-drug development stage and post-evaluation stage of listed drugs.

Human-induced pluripotent stem cell-derived cardiomyocytes (hiPSC-CMs)/human embryonic stem cell-derived cardiomyocytes (hESC-CMs) expressing cardiac-specific factors and structural proteins provide an alternative model for drug screening in vitro [5]. These cells are derived from human cells, have similar mechanical and electrical activities as adult cardiomyocytes, and are more reliable in evaluating cardiotoxicity than other cell lines [6]. Cardio non-labeled cell function analysis and culture system (Cardio-NLCS) combined hiPSC-CMs is a hybrid device supporting highly resolved impedance-based contractility measurements and electric field potential recordings that will become a more powerful and reliable means of detecting cardiotoxicity [2].

**Fig. 1:**
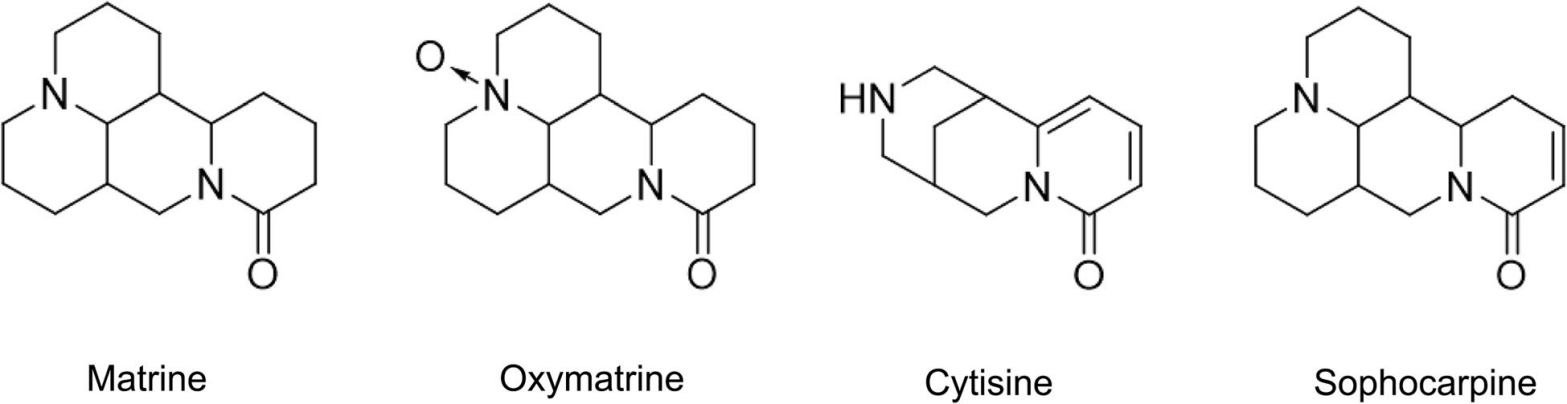
Chemical structure of *S. tonkinensis* alkaloid, matrine, oxymatrine, cytisine, and sophocarpine, respectively

*Sophora tonkinensis* Gapnep. (*S. tonkinensis*) is the dry root and rhizome of a leguminous plant, mainly distributed in Southern China and in the north of Vietnam [7]. The medicinal effect of *S. tonkinensis* was first published in “Kai Bao Materia Medica” of the ancient Chinese Tang Dynasty, which listed drugs with the main component of *S. tonkinensis*, for example, compound *S. tonkinensis* granules, compound *S. tonkinensis* injection, compound *S. tonkinensis* oral liquid, compound *S. tonkinensis* tablets were commonly used in the treatment of hepatitis B, pharyngitis, jaundice, arrhythmia, and others in modern clinical [8]. To date, the chemical components primarily consist of such compounds as alkaloids, flavonoids, and polysaccharides [9]. Pharmacological and toxicological studies have shown that *S. tonkinensis* has anti-tumor, liver-protecting, bacteriostatic, anti-inflammatory, immune-enhancing, anti-arrhythmia, and blood pressure lowering effects. However, this chemical can cause toxic side effects such as liver toxicity, neurotoxicity, cardiovascular toxicity, and gastrointestinal reactions [8]. In fact, liver toxicity has been extensively investigated, unlike cardiac toxicity, which has been researched less. The symptoms of severe cardiotoxicity clinically manifest as palpitations, increased heart rate, and decreased blood pressure. Ingredients of *S. tonkinensis* that are associated with toxicity mainly include matrine, oxymatrine, cytisine, and sophoridine. Their chemical structures were shown in Fig. 1 [9].

This article will conduct more in-depth research in the cardiotoxicity of matrine, oxymatrine, cytisine, and sophoridine. We detected impedance and potentially related indicators of hiPSC-CMs in real time, which could provide a basis for the study of the relationship among quantity-time-toxicity of *S. tonkinensis*, which could be greatly beneficial for promoting rational drug use in clinical practice. Drug-induced structural cardiotoxicity manifests morphological damage or loss of subcellular components of the heart leading to loss of contractility [10]. Therefore, measurement of structural cardiotoxicity including cell viability, oxidative stress, lactate dehydrogenase (LDH) leakage, and intracellular calcium is equally important for the comprehensive evaluation for cardiotoxicity of *S. tonkinensis* [11]. In conclusion, the contractile, electrophysiological, and structural cardiotoxicity of matrine, oxymatrine, cytisine, and sophocarpine was evaluated through human cardiomyocytes in order to provide guidelines for clinical medication management of *S. tonkinensis.*

## Methods

### Chemicals and reagents

Matrine, oxymatrine, cytisine, sophocarpine, and aconitine were purchased from Shanghai Winherb Medical Science Co., Ltd. (Shanghai, China) with purities higher than 98%. Cardiomyocyte recovery and maintaining medium were purchased from CELLAPYBIO (Beijing, China).

### Cell culture

hiPSC-CMs and GCAMP hESC-CMs were obtained from CELLAPYBIO (Cat# CA2201106, Cat# CA2206106, Beijing, China) and are a well-validated cell line. Briefly, each well of Nanion CardioExcyte 96 Sensor Plate (NSP-96) and corning-96 was pre-coated with 100 ml of a 1:100 diluted Matrigel solution (BD) and maintained at 37 °C overnight. The cells were thawed rapidly from liquid nitrogen into cardiomyocytes plating medium following recommended procedures and were incubated in maintaining medium at 37 °C, 5% CO_2_.

### Compound treatment

After plating for about 3 days, CMs matured and began to beat. At this time, different compounds were added, and experiments were performed 24 h after treatment. The experiments were grouped as follows: control group (normal medium); matrine, oxymatrine, sophocarpine, and cytisine treatment (2, 10, 50 μM) groups; and aconitine treatment (1 μM) group. The doses of toxicity studies above were determined by the effective therapeutic doses reported and included or exceeded the therapeutic dose [9, 12–14].

### Cardio non-labeled cell function analysis and culture system operation

Cardio-NLCS (Optoprobe Science LTD, Canada) is a turn-key system for efficient impedance and extracellular field potential (EFP) measurements of cell monolayers or tissue preparations. NSP-96 dedicated for Cardio-NLCS should be coated with BD and the wells should not run dry before adding the cell suspension. After seeding, the medium was not exchanged for 12 h. Subsequently, the medium exchange was completed once a day for 2–3 days and every 2 days thereafter. Adding compounds required a complete solution exchange to allow for exact dosing. After the cells were allowed to re-equilibrate for 2–3 h, the 2× concentrated compounds were added by exchanging half of the medium per well. In this experiment, several indexes (base impedance, amplitude, beat rate, and FPD) were recorded at the start of the experiment (control), then every 5 min for 4 h after compound application and every 30 min the same as cell growth period detection, lasting a minimum of 24 h. In this way, both time and concentration-dependent effects could be monitored. Impedance and EFP data analyses were taken every 4 h after compound administrations.

### Cell Counting Kit 8 assay

CMs were incubated in 10% CCK-8 (Dojindo, Kumamoto, Japan) that was diluted in normal culture medium at 37 °C until a visual color conversion occurred. The absorbance of each well was measured using a microplate reader (Spectrafluor, TECAN, Sunrise, Austria) at 450 nm [15]. Three individual experiments were performed. All samples were operated in duplicate.

### Typan blue exclusion assay

A 96-well plate was used to seed hiPSC-CMs at a concentration of 5 × 10^4^ cells per well. The cultured cells were treated with different concentrations of compounds and were incubated for another 48 h. CMs were digested with trypsin, and a small amount of cell suspension was added into the same amount of trypan blue solution. Countstar Automated Cell Counter IC1000 (Elite Life Sciences, Shanghai, China) was used to count the number of total and living cells. Data were presented as the mean ± SEM, *n* ≥ 3.

### LDH leakage assay

Cell treatment method was the same as above. After reaching the predetermined time, 120 μl of the supernatant of each well was taken for measuring LDH leakage using a commercially available kit (Beyotime Biotechnology, Shanghai, China) following the manufacturer’s instructions [16]. Data were presented as the mean ± SEM, *n* ≥ 3.

### Creatine kinase MB isoenzyme and cardiac troponin I ELISA assay

Cell treatment method was the same as above. After the administration, the cells were digested and then destroyed by repeated freeze-thaw and sonication to release the components in the cells. Centrifuged at 2500 rpm for 20 min, the supernatant was collected for the detection of creatine kinase MB isoenzyme (CK-MB) and cardiac troponin I (CTn-I) ELISA kit (Expandbiotech, Beijing, China) following the manufacturer’s instructions. Data were presented as the mean ± SEM, *n* ≥ 3.

### Superoxide dismutase, GSH, and malondialdehyde assay

Cell treatment was the same as discussed in the “Compound treatment” section. After reaching the predetermined time, the cells were washed with 4 °C physiological saline and later were added into the lysate and appropriately pipetted. After centrifuging at 1.2 × 10^4^ rpm for 5 min, the supernatant was obtained to measure the levels of SOD, GSH and MDA using a commercially available kit (Beyotime Biotechnology, Shanghai, China) following the manufacturer's instructions [17]. Data were presented as the mean ± SEM, *n* ≥ 3.

### Reactive oxygen species detection on hiPSC-CMs

A 96-well plate was used to seed hiPSC-CMs at a concentration of 5 × 10^4^ cells per well. The cultured cells were treated with different concentrations of compounds and were incubated for another 48 h. Cells were incubated in a maintaining medium containing 10 μM DCFH-DA (Beyotime Biotechnology, Shanghai, China) for 30 min at 37 °C in the dark [18]. The measurement of ROS was performed with IncuCyte™ S3 ZOOM cell imaging system (Essen BioScience, Ann Arbor, MI). TissueQuest 6.0 was used for the quantitative analysis of ROS formation. Data were presented as the mean ± SEM, *n* ≥ 3.

### Intracellular calcium on GCaMP hESC-CMs

The hESC-CMs in this experiment can express the GCaMP reporter gene by gene editing, and green fluorescence marker can be observed by a fluorescence microscope. The fluorescence intensity is proportional to Ca^2+^ concentration. The CMs were seeded into 96-well plates at 5000/well and were administered the same as above [19]. The fluorescence intensities of hESC-CMs were monitored using an IncuCyte™ S3 ZOOM cell imaging system (Essen BioScience, Ann Arbor, MI). Data were presented as the mean ± SEM, *n* ≥ 3.

#### Data analysis and statistics

All experiments were repeated at least three times. Data are expressed as the mean ± SD and analyzed by one-way ANOVA followed by Newman-Keuls multiple comparison test as appropriate (GraphPad Prism version 5 software). *P* < 0.05 was considered statistically significant.

## Results

### Optimization of hiPSC-CMs plating density on Cardio-NLCS

**Fig. 2:**
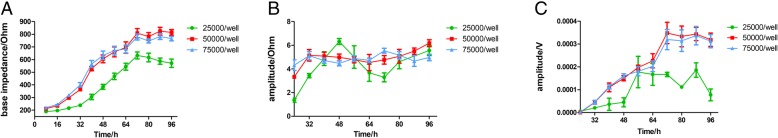
Optimization of hiPSC-CMs plating density on CardioExcyte 96. **a** Base impedance, **b** amplitude (IMP), and **c** amplitude (EFP) of hiPSC-CMs within 96 h after plating. Data are presented as the mean ± SEM, *n* ≥ 3

A preliminary test was used to determine the number of cells, as well as the time of administration. hiPSC-CMs were seeded at a density range of 25,000 to 75,000 cells per well on NSP-96. CMs were monitored for 96 h, and most began to beat 30 h after seeding. As shown in Fig. 2, the increase in the number of cells greatly promoted adhesion, and the number of cell seeded had a positive effect on the amplitude. Furthermore, 50,000 and 75,000 cells/well had no significant differences in base impedance and amplitude. Thus, the optimal plating density in this study was determined as 50,000 cells/well.

### Effect of matrine on the impedance and EFP signal in hiPSC-CMs

**Fig. 3:**
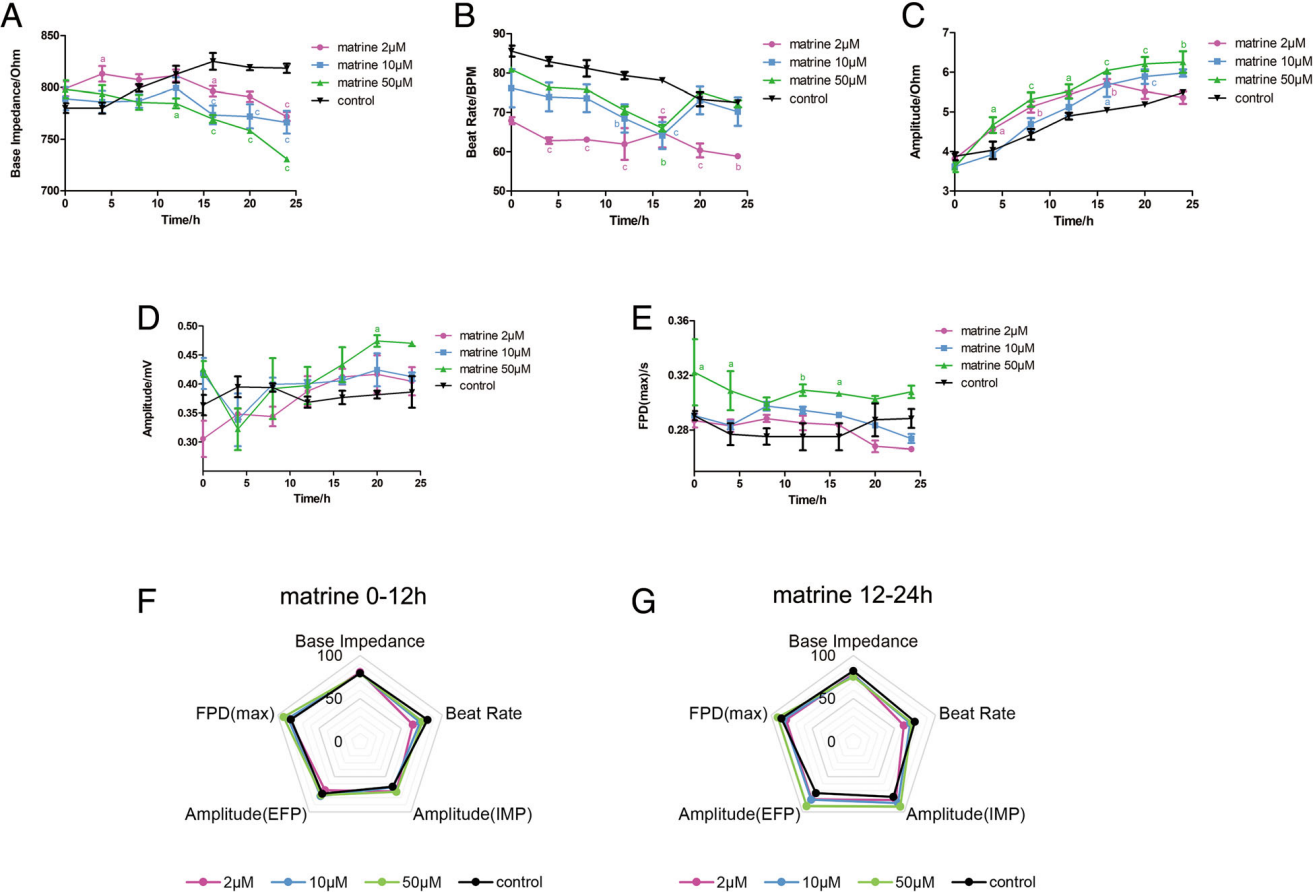
Effect of matrine on the impedance and EFP signal in hiPSC-CMs. The indicators include **a** base impedance, **b** beat rate, **c** amplitude of IMP, **d** amplitude of EFP, and **e** FPD (max). Comprehensive analysis of the above indicators is shown in spider charts in **f** 0–12 h and **g** 12–24 h. Data are presented as the mean ± SEM, *n* ≥ 3. ^a^*P* > 0.05, ^b^*P* > 0.01, and ^c^*P* > 0.001 vs the control group, and the colors of the superscript letters are the same with the broken lines

Matrine is one of the most important components in *S. tonkinensis*, and as such, its impedance and EFP signal were recorded by Cardio-NLCS. The base impedance and beat rate of the matrine groups are slightly lowered than that of the control group, while the other indicators showed an opposite trend in Fig. 3a, b. Both contractile and potential amplitude in Fig. 3c, d had an upward trend dependently. As shown in Fig. 3e, the FPD value of the high dose rose in the first half of matrine treatment. According to the comprehensive analysis results of the radar chart in Fig. 3f, g, the change of the beat rate on the first half of administration was larger, and the differences of amplitude in different groups gradually increased with the advancement of the experiment.

### Effect of oxymatrine on the impedance and EFP signal in hiPSC-CMs

**Fig. 4:**
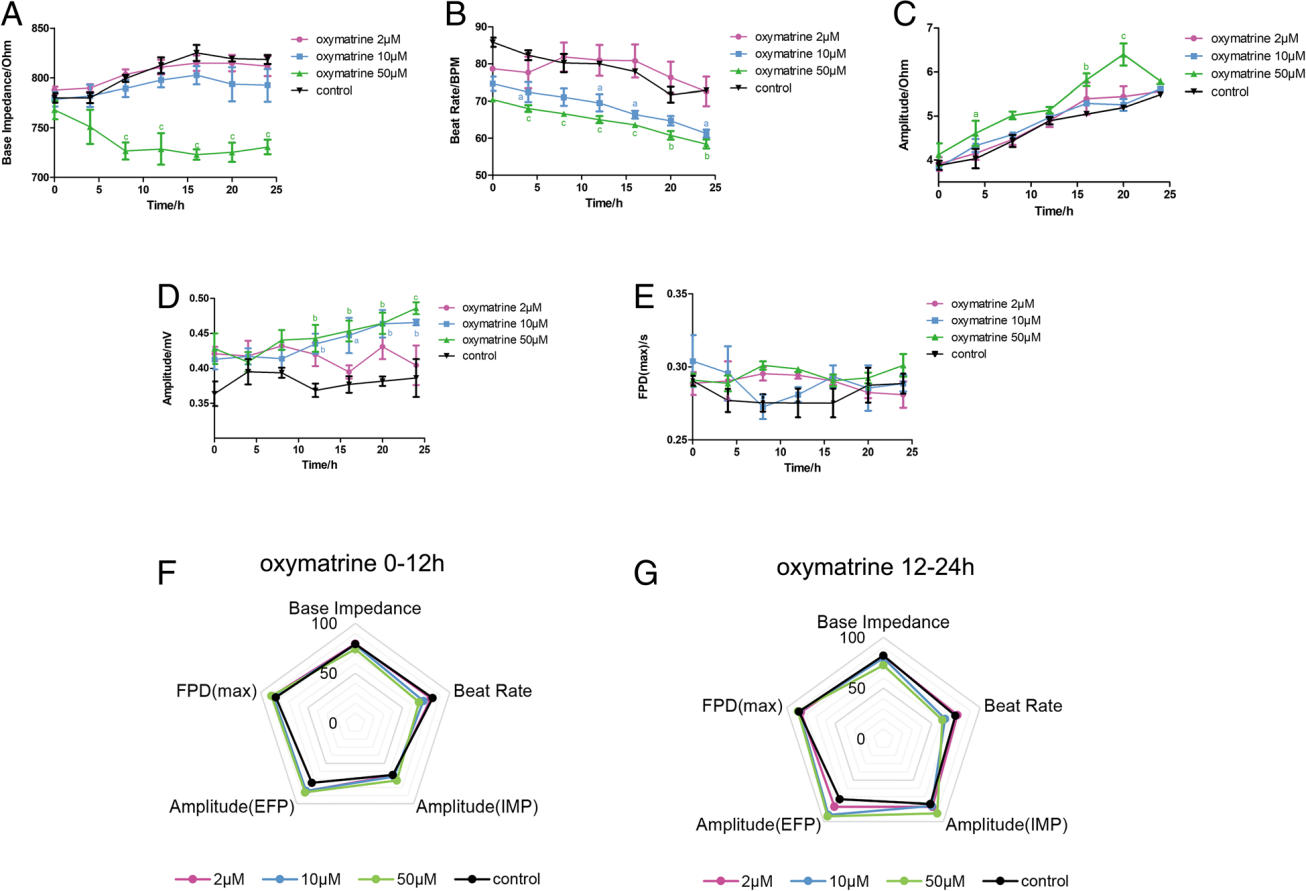
Effect of oxymatrine on the impedance and EFP signal in hiPSC-CMs. The indicators include **a** base impedance, **b** beat rate, **c** amplitude of IMP, **d** amplitude of EFP, and **e** FPD (max). Comprehensive analysis of the above indicators is shown in spider charts in **f** 0–12 h and **g** 12–24 h. Data are presented as the mean ± SEM, *n* ≥ 3. ^a^*P* > 0.05, ^b^*P* > 0.01, and ^c^*P* > 0.001 vs the control group, and the colors of the superscript letters are the same with the broken lines

Oxymatrine is another major alkaloid component in *S. tonkinensis*. The base impedance in Fig. 4a showed no change, except 10 μM oxymatrine treatment and the beat rate of the oxymatrine groups in Fig. 4b were slightly lower than the control group. Both contractile and potential amplitude of Fig. 4c, d had an upward dose-dependent trend. FPD value in Fig. 4e remained stable in all groups. According to the comprehensive analysis results of the radar chart in Fig. 4f, g, the changes of the beat rate and contractile amplitude on the first half of administration were larger, and the differences of amplitude (IMP, EFP) in different groups gradually increase with the advancement of the experiment. However, FPD value had no significant change among groups over time.

### Effect of cytisine on the impedance and EFP signal in hiPSC-CMs

**Fig. 5:**
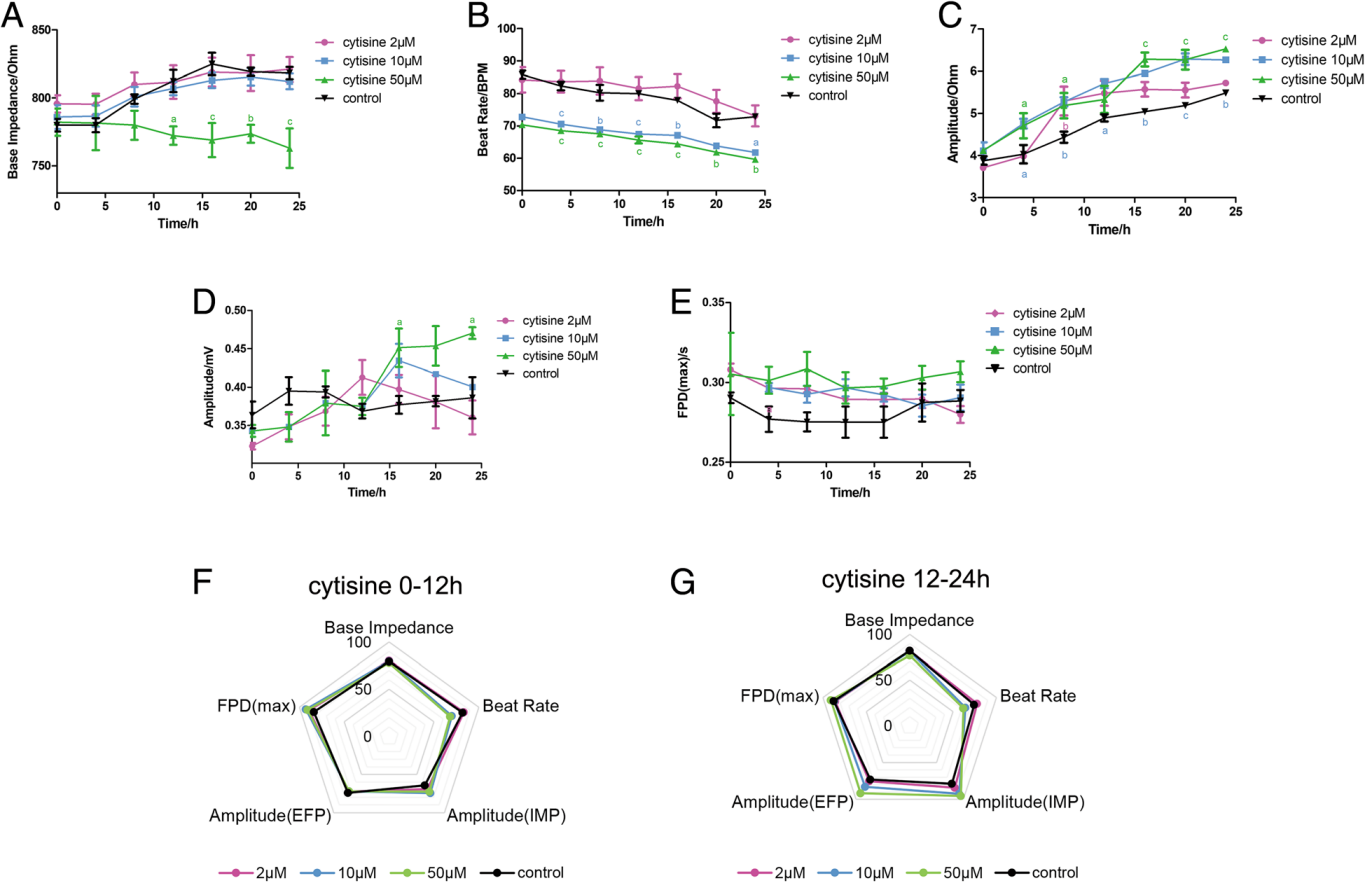
Effect of cytisine on the impedance and EFP signal in hiPSC-CMs. The indicators include **a** base impedance, **b** beat rate, **c** amplitude of IMP, **d** amplitude of EFP, and **e** FPD (max). Comprehensive analysis of the above indicators is shown in spider charts in **f** 0–12 h and **g** 12–24 h. Data are presented as the mean ± SEM, *n* ≥ 3. ^a^*P* > 0.05, ^b^*P* > 0.01, and ^c^*P* > 0.001 vs the control group, and the colors of the superscript letters are the same with the broken lines

Cytisine is also an alkaloid extracted from *S. tonkinensis*. The base impedance in Fig. 5a showed no change except 50 μM cytisine treatment and the beat rate of the cytisine groups (10, 50 μM) in Fig. 5b were slightly lower than the control group. Both contractile and potential amplitudes are shown in Fig. 5c, d had an upward trend dependently. FPD value in Fig. 5e remained stable in all groups. According to the comprehensive analysis results of the radar chart in Fig. 5f, g, the changes of the beat rate and contractile amplitude on the first half of administration were larger, and the differences of potential amplitude and FPD in different groups gradually increase with the advancement of the experiment.

### Effect of sophocarpine on the EFP signal in hiPSC-CMs

**Fig. 6:**
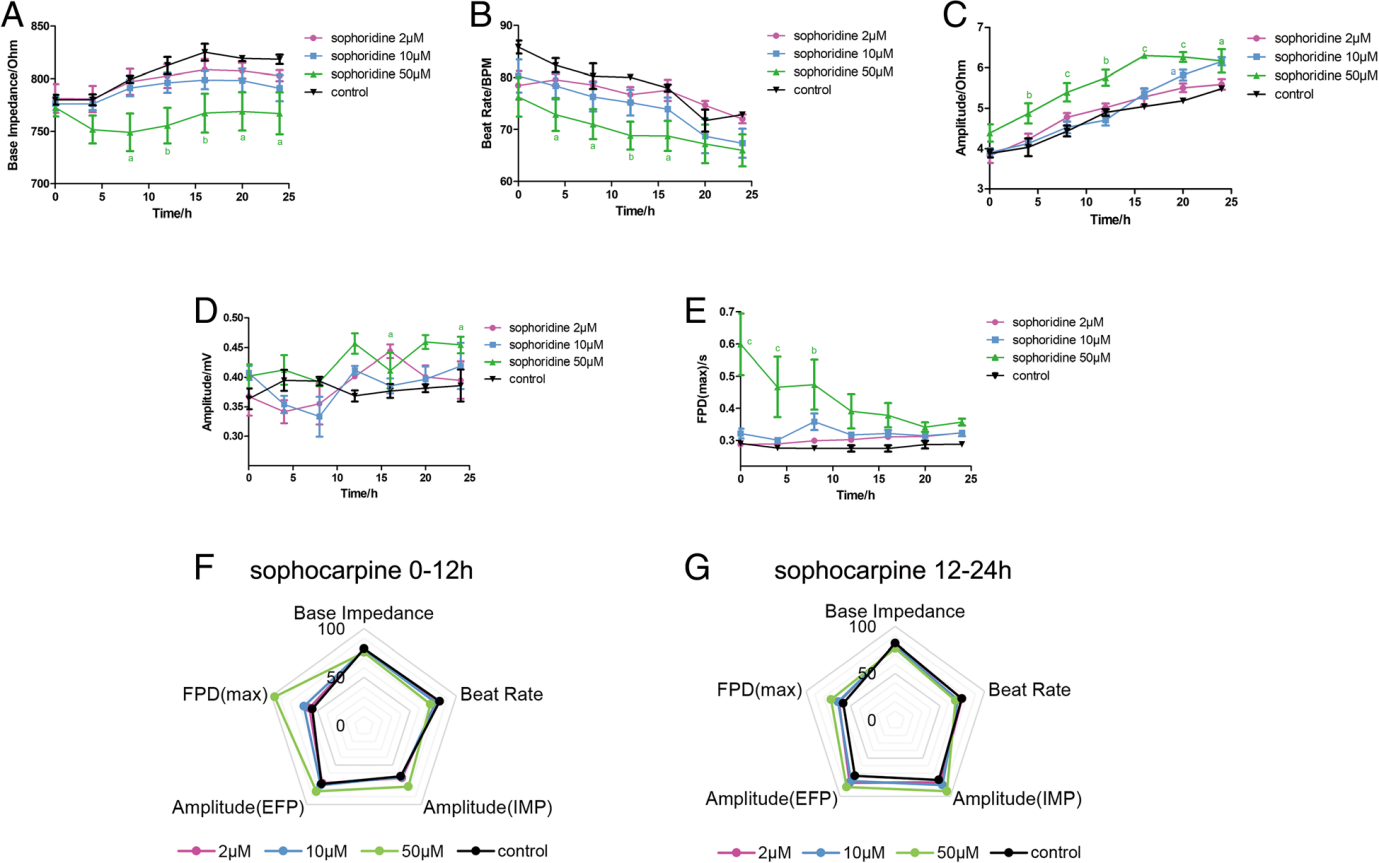
Effect of sophocarpine on the impedance and EFP signal in hiPSC-CMs. The indicators include **a** base impedance, **b** beat rate, **c** amplitude of IMP, **d** amplitude of EFP, and **e** FPD (max). Comprehensive analysis of the above indicators is shown in spider charts in **f** 0–12 h and **g** 12–24 h. Data are presented as the mean ± SEM, *n* ≥ 3. ^a^*P* > 0.05, ^b^*P* > 0.01, and ^c^*P* > 0.001 vs the control group, and the colors of the superscript letters are the same with the broken lines

Sophocarpine is also an alkaloid extracted from *S. tonkinensis*. The base impedance and potential amplitudes in Fig. 6a, d showed no change, and the beat rate of the cytisine groups in Fig. 6b was slightly lowered compared to the control group. The contractile amplitude in Fig. 6c had an upward dose-dependent trend. FPD value showed large differences in the first 12 h and remained stable after 15 h of treatment. According to the comprehensive analysis results of the radar chart in Fig. 6f, g, the changes of the beat rate, potential amplitude, and FPD on the first half of administration were larger, and the differences of potential amplitude in different groups gradually increase with the advancement of the experiment.

### Effect of matrine, oxymatrine, cytisine, and sophocarpine on cell viability and cell cytotoxicity in hiPSC-CMs

**Fig. 7:**
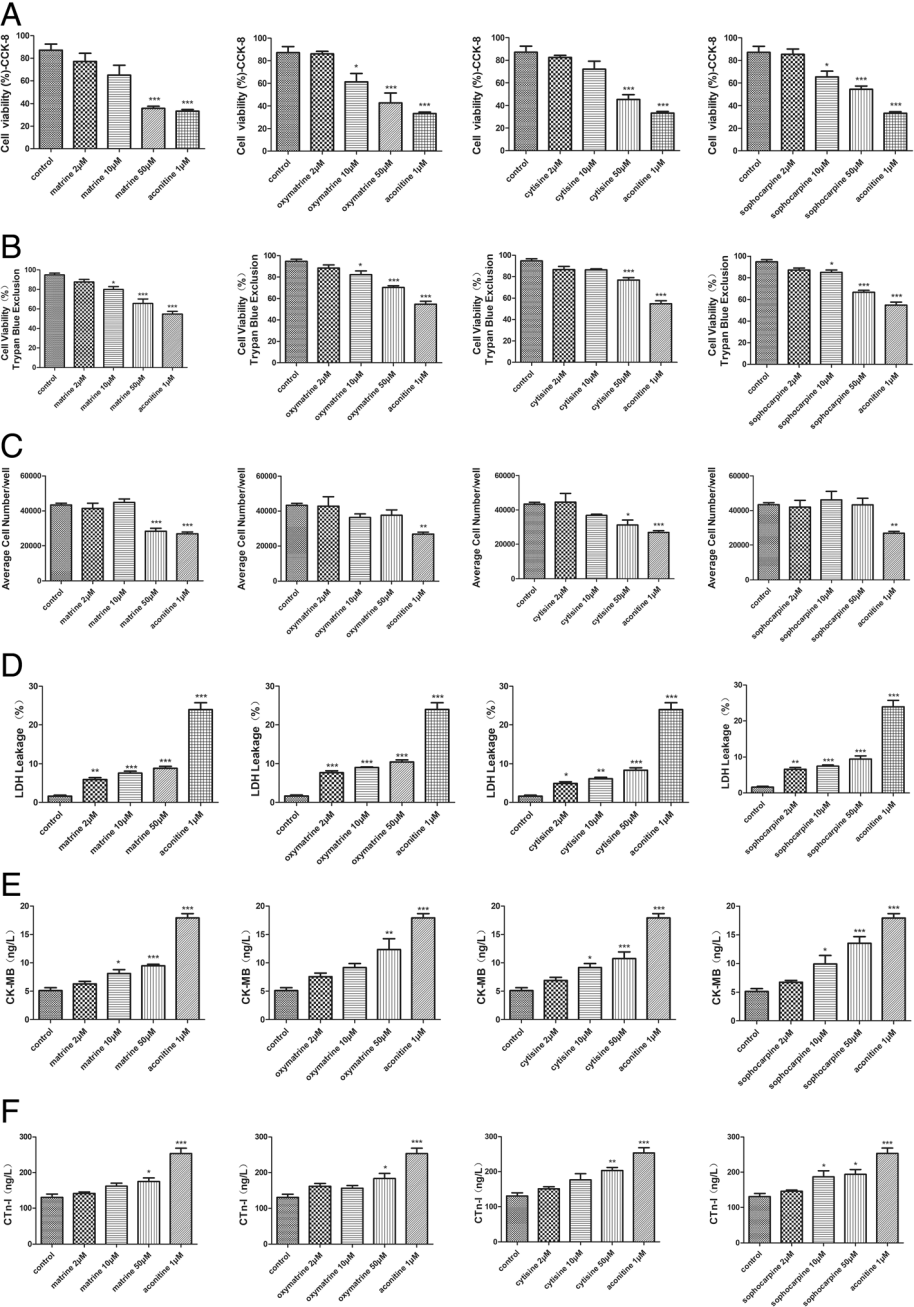
Effect of matrine, oxymatrine, cytisine, and sophocarpine on cell viability and cell cytotoxicity in hiPSC-CMs. **a** Cell Counting Kit 8 (CCK-8) and **b**, **c** trypan blue exclusion assay were used to determine the cell viability, and **d** LDH leakage and the level of **e** CK-MB and **f** CTnI were used to determine cell damage and cytotoxicity. Data are presented as the mean ± SEM, *n* ≥ 3. *P > 0.05, **P > 0.01, and ***P > 0.001 vs the control group

The cell viability was evaluated using Cell Counting Kit 8 (CCK-8) and trypan blue exclusion assay [20], and hiPSC-CMs were incubated with different concentrations of matrine, oxymatrine, cytisine, and sophocarpine (2, 10, 50 μM) for 24 h (Additional file 1). The results showed cell viability decreased dose-dependently with increasing dose concentrations, particularly cell viability was significantly decreased at the 10 μM dose in Fig. 7a–c.

Furthermore, membrane damage that leads to the increase of LDH, CK-MB, and CTnI is generally considered irreversible, so the three indicators reflected cell cytotoxicity to some extent [21]. As shown in Fig. 7d–f, aconitine induced a large amount of LDH, CK-MB, and CTnI in the supernatant, and the addition of four compounds also led to higher LDH, CK-MB, and CTnI levels in a dose-dependent fashion. The results were consistent with CCK-8 and trypan blue exclusion assay, which indicated medium and high doses of four compounds elicited cardiotoxicity.

### Effect of matrine, oxymatrine, cytisine, and sophocarpine on oxidative stress in hiPSC-CMs

**Fig. 8:**
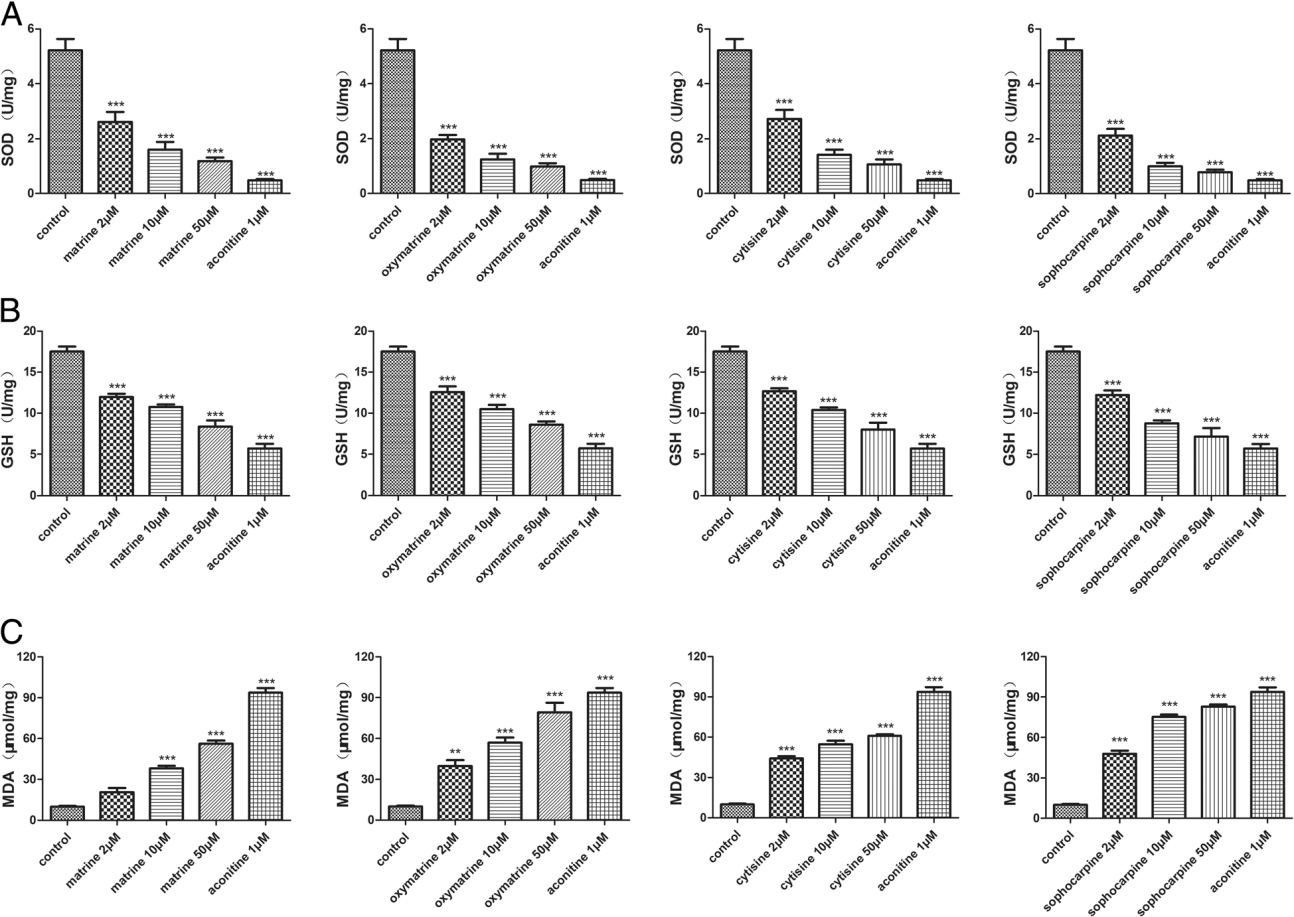
Effect of matrine, oxymatrine, cytisine, and sophocarpine on oxidative stress in hiPSC-CMs. **a** SOD activity. **b** GSH activity. **c** MDA content. Data are presented as the mean ± SEM, *n* ≥ 3. *P > 0.05, **P > 0.01, and ***P > 0.001 vs the control group

SOD and GSH are antioxidase, and MDA is a lipid peroxidation product. The three indicators can reflect the level of oxidative damage [22, 23]. In the present study, the enzyme activities of SOD and GSH sharply lowered after treatment of matrine, oxymatrine, cytisine, and sophocarpine dose-dependently, but the level of MDA rose in Fig. 8. The results indicated that the four compounds could impair oxidative stability and increase lipid peroxidation.

**Fig. 9:**
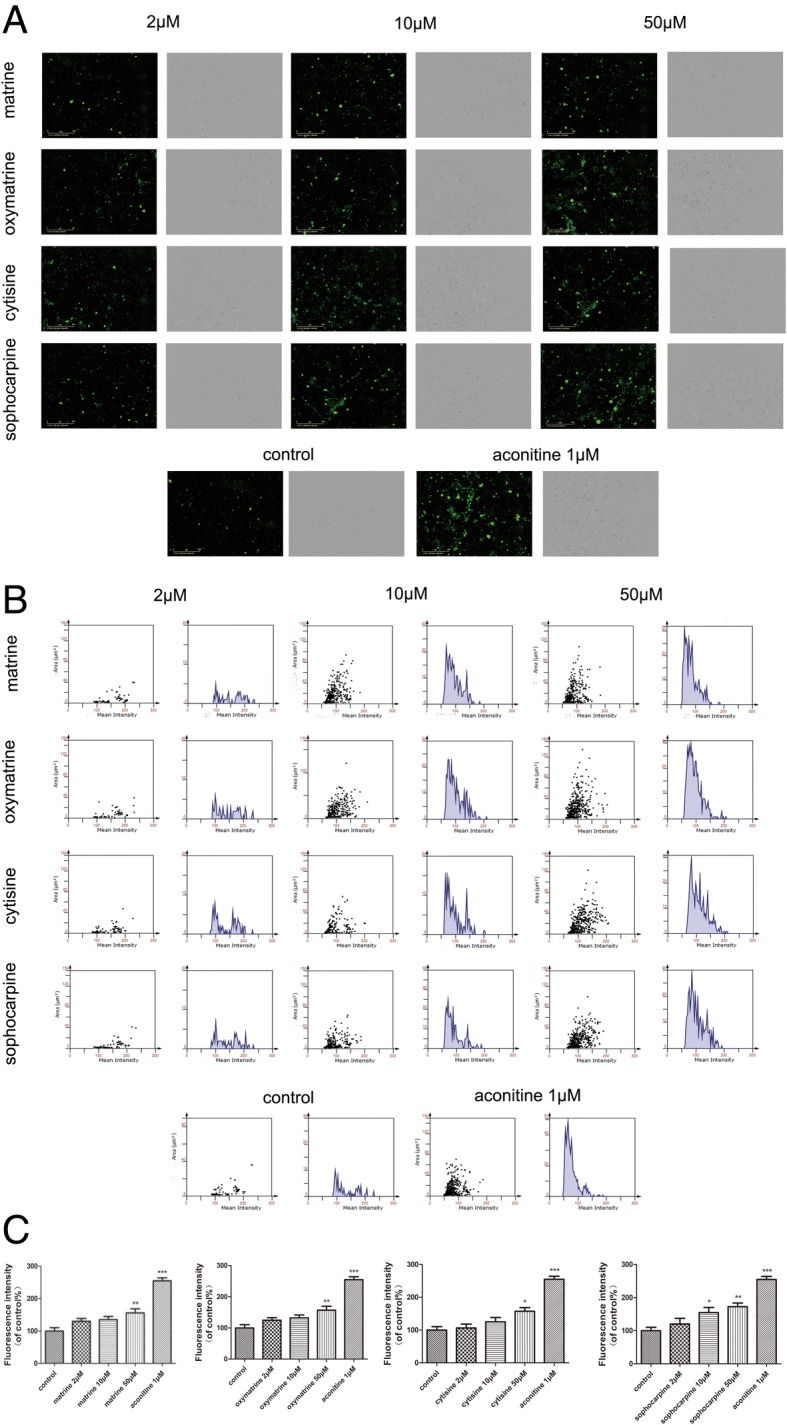
Effect of matrine, oxymatrine, cytisine, and sophocarpine on ROS formation in hiPSC-CMs. After treatment with matrine, oxymatrine, cytisine, and sophocarpine, **a** the images of ROS fluorescence and bright field were acquired intuitively using IncuCyte™ S3 ZOOM cell imaging system and **b**, **c** the fluorescence intensity was analyzed quantitatively using TissueQuest 6.0. The scale bar is 400 μm. Data are presented as the mean ± SEM, *n* ≥ 3. *P > 0.05, **P > 0.01, and ***P > 0.001 vs the control group

Besides, the generation of intracellular ROS could induce oxidative damage, and DCFH-DA is generally used to measure ROS generation in cells [24]. The fluorescence images showed that there was a visible increase of the fluorescence staining of ROS (green) in the CMs after treating the high dose of four compounds, the same as statistical results of fluorescence density as shown in Fig. 9.

### Effect of matrine, oxymatrine, cytisine, and sophocarpine on intracellular calcium in hESC-CMs with GFP

**Fig. 10:**
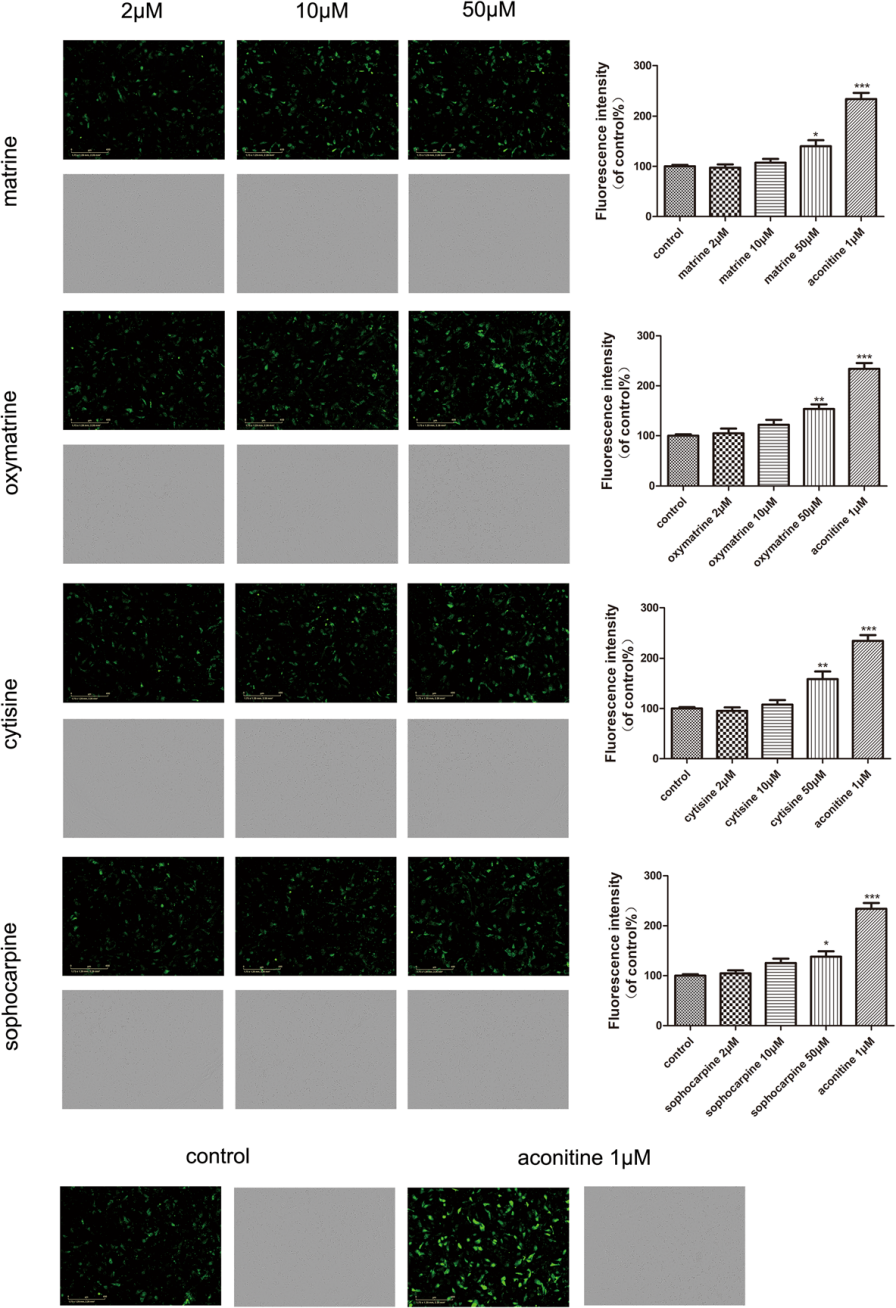
Effect of matrine, oxymatrine, cytisine, and sophocarpine on intracellular calcium in hESC-CMs. After treatment of matrine, oxymatrine, cytisine, and sophocarpine, the images of calcium fluorescence and bright field were acquired intuitively and the fluorescence intensity was analyzed quantitatively using IncuCyte™ S3 ZOOM cell imaging system. The scale bar is 400 μm. Data are presented as the mean ± SEM, *n* ≥ 3. *P > 0.05, **P > 0.01, and ***P > 0.001 vs the control group

Intracellular Ca^2+^ accumulation is thought to initiate myocardial injury and impair contractile function. The CMs in this experiment can generate green fluorescence protein (GFP) and were not easy to quench [25, 26]. The images from Fig. 10 showed the fluorescence intensity of calcium increased drastically after aconitine treatment, and only high dose of other compounds started to increase the fluorescence intensity significantly.

## Discussion

Early and effective cardiac safety pharmacology evaluation is important to guarantee measures for advancing the candidate drug [27]. The Comprehensive in vitro Proarrhythmia Assay (CiPA) was established to develop a new paradigm for assessing proarrhythmic risk after the two guidelines ICH S7B and ICH E14, early single cardiac safety assessment methods [3, 4, 28]. This new strategy represented a shift in the pharmacology evaluation model of cardiac safety, which replaced the previous method of assessing cardiac safety using single parameters (hERG) and indirect indicators (QT prolongation) [5, 29], prone to false positives or false negatives. For example, phenobarbital led to QT prolongation without causing a fatal arrhythmia. Historically, effects on cardiac contractility were evaluated in animal studies to determine the suitability of a candidate drug to continue on a development path [30]. Recently, the use of hiPSC-CMs based on CiPA could avoid species differences and evaluate the effects of drugs on cardiac function and structure, thereby allowing a physiologically accurate dissection of the adverse action on drugs [31, 32]. We detected cardiac contractility and some features about EFP by Cardio-NLCS, such as field potential duration (FPD)—which is analogous to the QT interval in vivo—and torsadogenic risk based on observations of early afterdepolarization and triggered activity, providing a more comprehensive assessment of cardiac function and toxicity. The combination of Cardio-NLCS and hiPSC-CMs better reflects vivo cardiotoxicity testing by offering relevant data for in vitro recordings from intact, synchronously beating networks of cardiomyocytes.

In this study, we evaluated the potential use of hiPSC-CMs for *S. tonkinensis*-mediated cardiotoxicity in humans by investigating some functional indicators [33]. The clinical application of *S. tonkinensis* is extensive, including chronic hepatitis B, arrhythmia, and gastric ulcer, but there are few studies on its cardiotoxicity and its mechanism. To ensure the safety of medication of *S. tonkinensis*, we selected its main toxic component, matrine, oxymatrine, cytisine, and sophocarpine, and studied their cardiac safety to provide some guidance for clinical rational drug use. In Cardio-NLCS, the EFP reflects the population action potential, hence the crossing of ions over the cell membrane, while the impedance reflects the contraction, or the intracellular calcium flux determining the duration and strength of contraction. The four components did not change much in the base impedance relative to the control group, except for the high-dose group, the resistance decreased significantly. Our data showed that matrine affected the contraction and EFP of CMs and caused a concentration-dependent decrease in amplitude both impedance and EFP. Previous research demonstrated that the therapeutic dose of matrine and oxymatrine were able to act as hERG channel activators to suppress long QT syndrome (LQTS) [8]. The effect on FPD, positive correlation with QT interval, was greater within 16 h after matrine administration, indicating that excessive dose of matrine also led to QT prolongation and the effect of matrine on potential was rapidly produced after administration. Several studies have shown that matrine had a positive inotropic effect and a negative frequency effect on the heart [34, 35]. For beat rate, at low, therapeutically relevant concentrations (2 μM) of matrine, a decrease in the beat rate can be detected, while at higher concentrations (10, 50 μM), an increase in beat rate can be detected in comparison with the low-dose group, indicating negative frequency effect of matrine and increased cardiotoxicity with dose increasing. Oxymatrine was reported to have a cardioprotective effect against doxorubicin-induced cardiotoxicity by inhibiting apoptosis and oxidative stress [12]. However, in our study, oxymatrine had a large fluctuation in the amplitude (EFP) and beat rate dose-independently with a similar trend to matrine, indicating its cardiotoxicity mainly expressed by potential and pulsation. Cytisine had a greater impact on contraction and amplitude (EFP); however, it had no significant effect on FPD similar to oxymatrine, implying that both alkaloids have little effect on QT interval. Sophocarpine was another alkaloid component that had a significant impact on both impedance and EFP. A high dose (50 μM) of sophocarpine showed significant differences in each indicator compared other groups, especially FPD. FPD immediately increased in a jump after high-dose (50 μM) administration sophocarpine, and the gap gradually narrowed as the administration time increased, and there was no significant difference from the other groups after 24 h of administration, which indicated sophocarpine-induced cardiotoxicity on EFP produced quickly fast but lasted for a short time.

Several structural and mechanism-related indicators also collectively indicated the degree of cardiotoxicity. Cell viability using CCK-8 and trypan blue exclusion assay reduced with increasing concentrations of four alkaloids, and the high concentration of them and aconitine, the positive drug showed a great significance. The concentration of LDH and CK-MB in cellular supernatant reflected the degree of cell membrane and integrity and also served as the diagnostic markers of myocardial damage [36]. In our experiment, four compounds increased the level of LDH and CK-MB dose-dependently and aconitine increased immensely the level of LDH and CK-MB. An additional cellular assay that could be useful to further understand the cellular cytotoxicity is the level of ROS, lipid accumulation, and antioxidant enzymes. SOD is a metalloprotein and accomplishes its antioxidant functions by enzymatically detoxifying the peroxides and superoxide anion, and GSH is an ubiquitous sulfhydryl-containing molecule in cells that is responsible for maintaining cellular oxidation-reduction homeostasis [17]. Changes in GSH homeostasis can be monitored as an indication of cell damage [23]. In the study, the activities of two antioxidant enzymes both decreased drastically with dose dependence. However, we found that the level of MDA, which is one of the several low-molecular-weight end products formed via the decomposition of certain primary and secondary lipid peroxidation products, dose-dependently increased after administration. Several physiological functions, including cell survival, growth, differentiation, and metabolism, are mediated by tightly regulated low levels of ROS. However, the presence of ROS is a double-edged sword, and excessive production of ROS can damage macromolecules, including DNA, proteins, and lipids [37]. Thus, aberrant ROS generation constitutes a major mechanism of pathological cell death. Conforming to the predicted results, the fluorescence intensity of ROS in the high-dose groups elevated compared with the control group, which is consistent with the above results, indicating that the CMs were toxic after high-dose administration. It was reported that matrine-type alkaloids had protective effects by inhibiting excessive ROS production in vivo [38]; however, our results showed matrine and others induced ROS production in excess when the dose was sufficiently high and aconitine also induced excessive ROS production. The above results indicated that *S. tonkinensis*-induced cardiomyocyte toxicity was associated with oxidative stress.

Drug-induced long QT is most commonly induced by affecting the rapid potassium current Ikr by binding to the hERG ion channel. In addition to potassium channels, the activity of myocardial cells is largely dependent on sodium and calcium channels, whereas activation of sodium channels is required for the generation of an action potential, inward Ca^2+^ currents through (L-type) calcium channels counterbalance outward potassium currents in the repolarization phase [39]. Therefore, intracellular calcium plays a key role in maintaining cardiac excitation-contraction coupling. The intracellular Ca^2+^ signal was determined by calcium fluorescence intensity. It was previously reported that matrine significantly attenuated the inhibition of [Ca^2+^]_*i*_ by homocysteine in the isolated heart [35] and upregulated I_Ca-L_ density and α1C/Cav1.2 expression in mice [40]. In our study, the images in Fig. 10 showed the intensity of calcium increased significantly after high-dose (50 μM) treatment, which showed the production of calcium overload and proved matrine and other three alkaloids could increase [Ca^2+^]_*i*_ on hiPSC-CMs.

**Fig. 11:**
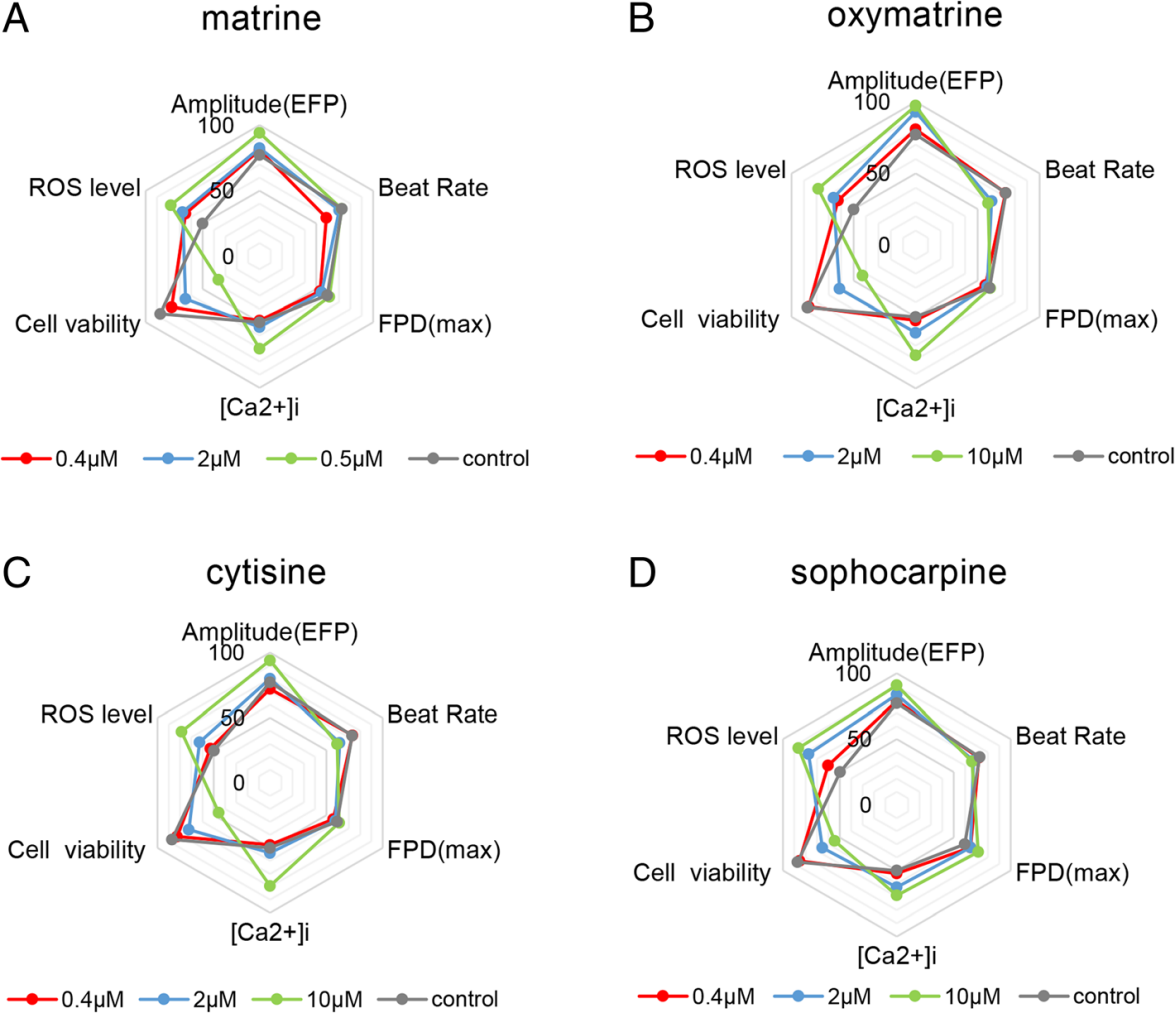
Comprehensive analysis of **a** matrine, **b** oxymatrine, **c** cytisine, and **d** sophocarpine on amplitude (EFP), beat rate, FPD (max), [Ca^2+^]_*i*_, cell viability, and ROS level in spider charts. All indicators were collected 24 h after administration

*S. tonkinensis*-induced cardiotoxicity is related to functional and structural changes of cardiomyocytes, and four alkaloids in this study showed different sensitivity to various indicators as shown in Table 1. For functional assessment, matrine and sophocarpine had significance on both impedance and EFP, and matrine focused more on impedance and sophocarpine did EFP; oxymatrine and cytisine significantly affected the impedance. Structural changes were also significant with dose dependence, especially cell viability and LDH leakage in Table 1 and Fig. 11. In addition, the spider charts in Fig. 11 clearly showed that the structural damage synchronized with functional changes, implying there were close internal connections between the two aspects. However, the specific link between the two needs further research in the future to clarify.

**Table 1:** Conclusive analysis of matrine, oxymatrine, cytisine, and sophocarpine on contractile, electrophysiological, and structural cardiotoxicity

Compounds	Indicators								
	Functional assessment					Structural cardiotoxicity			
	Base impedance	Beat rate	Amplitude (IMP)	Amplitude (EFP)	FPD	Cell viability (μM)	ROS level (μM)	[Ca^2+^]_*i*_ (μM)	LDH leakage (μM)
Matrine	10 μM (16 h)	2 μM	10 μM (4 h)	50 μM (20 h)	50 μM	50	50	50	2
Oxymatrine	50 μM (8 h)	10 μM (4 h)	50 μM (4 h)	10 μM (12 h)	No effect	10	50	50	2
Cytisine	50 μM (12 h)	10 μM	10 μM (4 h)	50 μM (16 h)	No effect	50	50	50	2
Sophocarpine	50 μM (8 h)	50 μM (4 h )	50 μM	50 μM (16 h)	50 μM (0–12 h)	10	10	50	2

The data in the chart was the lowest concentration (and initial time or periods of time) that demonstrated a significant change compare with the control group in these indicators

A limitation is that sample size in our study is a little low. But we used at least three samples for statistical analysis and found some interesting results. Therefore, the next step is to verify the results that have been completed by expanding the sample size, and further study will be carried out to gain a deeper understanding of the mechanism of cardiotoxicity.

## Conclusions

In summary, our study indicated that matrine, oxymatrine, cytisine, and sophocarpine could induce cardiotoxicity through changing the cardiac impulse function or/and extracellular field potential of cardiomyocytes related to oxidative stress and disruption of calcium homeostasis, but the influence of each component on the contractile function and field potential was inconsistent. Mechanism and specific targets will be investigated in our further research, aiming at providing a basis for the clinical dose of *S. tonkinensis* and avoiding unnecessary toxic side effects effectively. In addition, this study used an efficient and effective approach to study cardiotoxicity, which is also helpful to evaluate the cardiotoxicity of listed drugs and novel drug candidates.

## Additional file

Additional file 1: **Figure S1.** Effect of matrine, oxymatrine, cytisine, and sophocarpine on TUNEL staining in hiPSC-CMs. After treatment with matrine, oxymatrine, cytisine, and sophocarpine, the images of TUNEL fluorescence and bright field were acquired intuitively using IncuCyte™ S3 ZOOM cell imaging system and the fluorescence intensity was analyzed quantitatively using TissueQuest 6.0. (A) The fluorescence images showed that there was a huge increase of the fluorescence staining (green) in the CMs after treating the high dose of four compounds and aconitine, (B-C) the same as flow graphs and statistics, which indicated that high dose of *S. tonkinensis* induced apoptosis. The scale bar is 400 μm. Data are presented as the mean ± SEM, *n* ≥ 3. *P > 0.05, **P > 0.01, and ***P > 0.001 vs the control group. (TIF 23588 kb)

## Abbreviations

Cardio-NLCS: Cardio non-labeled cell function analysis and culture system
CCK-8: Cell Counting Kit 8
CiPA: Comprehensive in vitro Proarrhythmia Assay
CK-MB: Creatine kinase MB isoenzyme
CTn-I: Cardiac troponin I
EFP: Extracellular field potential
FPD: Field potential duration
GFP: Green fluorescence protein
hESC-CMs: Human embryonic stem cell-derived cardiomyocytes
hiPSC-CMs: Human-induced pluripotent stem cell-derived cardiomyocytes
LDH: Lactate dehydrogenase
LQTS: Long QT syndrome
MDA: Malondialdehyde
MEA: Multi-electrode arrays
NSP-96: Nanion CardioExcyte 96 Sensor Plate
ROS: Reactive oxygen species
*S. tonkinensis*: *Sophora tonkinensis* Gapnep.
SOD: Superoxide dismutase
TdP: Torsade de pointes
