# Correction: An integrated characterization of contractile, electrophysiological and structural cardiotoxicity of *Sophora tonkinensis* Gapnep. in human pluripotent stem cell-derived cardiomyocytes

**DOI:** 10.1186/s13287-023-03540-z

**Published:** 2023-10-31

**Authors:** Ruiying Wang, Min Wang, Shan Wang, Ke Yang, Ping Zhou, Xueheng Xie, Qi Cheng, Jingxue Ye, Guibo Sun, Xiaobo Sun

**Affiliations:** 1 Key Laboratory of Bioactive Substances and Resources Utilization of Chinese Herbal Medicine, Ministry of Education, Institute of Medicinal Plant Development, Chinese Academy of Medical Sciences & Peking Union Medical College, Beijing, 100193 China; 2 Collaborative Innovation Center of Yangtze River Delta Region Green Pharmaceuticals, Zhejiang University of Technology, No. 18, Chaowang Road, Xiacheng District, Hangzhou, 310014 Zhejiang China; 3 Harbin University of Commerce, Harbin, 150028 Heilongjiang China; 4 Beijing Health Olight Technology Co., Ltd, Beijing, 100068 China

**Correction: Stem Cell Research & Therapy (2019) 10:20** 10.1186/s13287-018-1126-4

In the original article [1], the authors identified that Fig. 9 and Fig. 10 in the article used the wrong images because of incorrect figure assembly.

In Fig. 9A, the ROS fluorescence image of sophocarpine-10 μM was the same as that of cytisine-50 μM. The authors identified that the image of cytisine-50 μM was uploaded incorrectly, and the correct ROS fluorescence and bright field image of cytisine-50 μM in reassembled Fig. 9 were provided.

In Fig. 10, the intracellular calcium fluorescence image of matrine-10 μM was the same as that of matrine-50 μM. The authors identified that the image of matrine-10 μM was uploaded incorrectly, and the correct calcium fluorescence and bright field image of matrine-10 μM in the reassembled Fig. 10 were provided.

The revised Figs. 9 and 10 were given in this article.

**Fig. 9:**
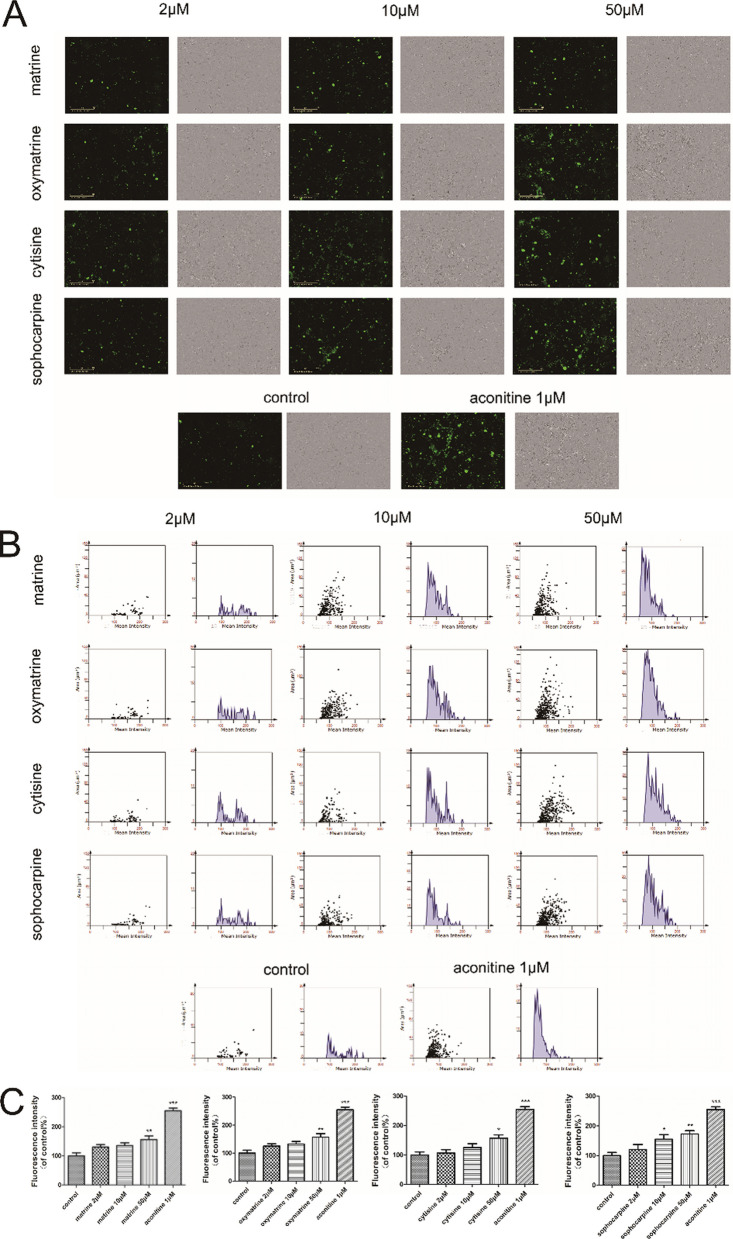
Effect of matrine, oxymatrine, cytisine and sophocarpine on ROS formation in hiPSC-CMs. After treatment with matrine, oxymatrine, cytisine and sophocarpine, **A** the images of ROS fluorescence and bright field were acquired intuitively using IncuCyte™ S3 ZOOM cell imaging system and **B**, **C** the fluorescence intensity was analysed quantitatively using TissueQuest 6.0. The scale bar is 400 μm. Data are presented as the mean ± SEM, n ≥ 3. *P > 0.05, **P > 0.01 and ***P > 0.001 *vs* control group.

**Fig. 10:**
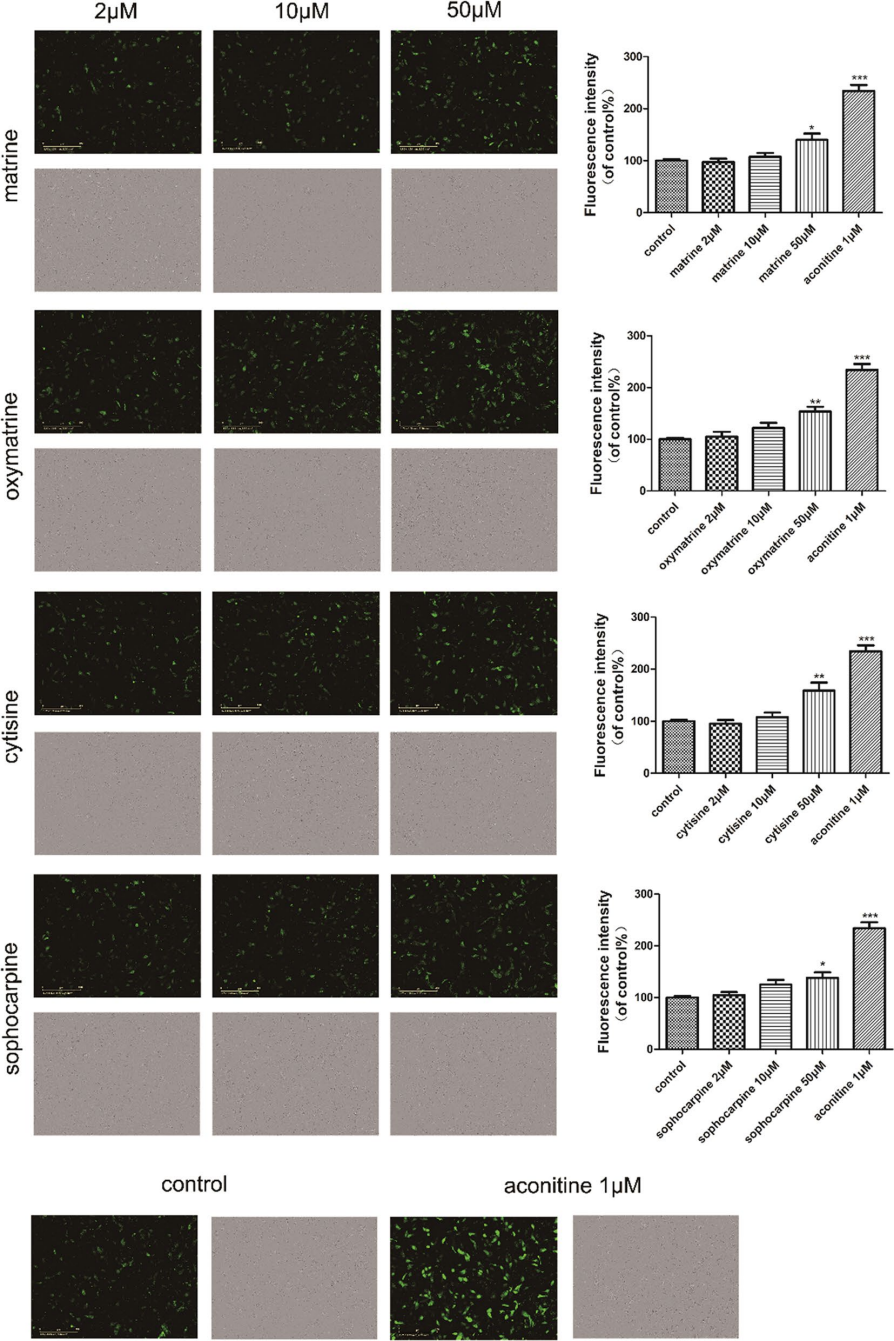
Effect of matrine, oxymatrine, cytisine and sophocarpine on intracellular calcium in hESC-CMs. After treatment of matrine, oxymatrine, cytisine and sophocarpine, the images of calcium fluorescence and bright field were acquired intuitively and the fluorescence intensity was analysed quantitatively using IncuCyte™ S3 ZOOM cell imaging system. The scale bar is 400 μm. Data are presented as the mean ± SEM, n ≥ 3. *P > 0.05, **P > 0.01 and ***P > 0.001 *vs* control group

Although this correction does not affect conclusions of article, the authors still apologize to the editor and the readership of the journal for carelessness in figure assembly.
