# Supplementary figures and images for: An integrated characterization of contractile, electrophysiological, and structural cardiotoxicity of Sophora tonkinensis Gapnep. in human pluripotent stem cell-derived cardiomyocytes
Source: Stem Cell Res Ther. 2019 Jan 11;10:20. doi: 10.1186/s13287-018-1126-4 (PMC6330446; doi:10.1186/s13287-018-1126-4)

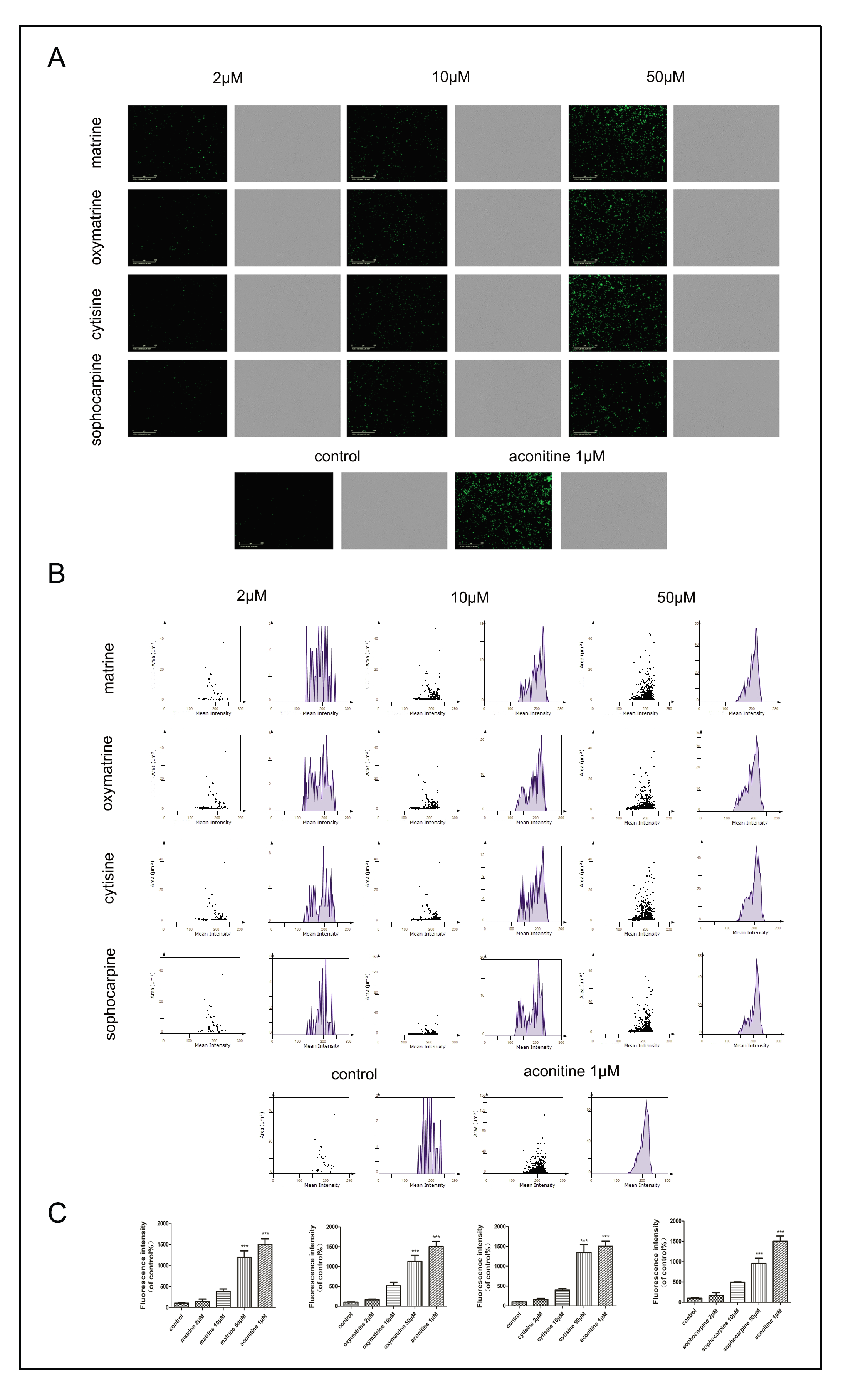

Supplement: Supplementary file 1 — Figure S1. Effect of matrine, oxymatrine, cytisine, and sophocarpine on TUNEL staining in hiPSC-CMs. After treatment with matrine, oxymatrine, cytisine, and sophocarpine, the images of TUNEL fluorescence and bright field were acquired intuitively using IncuCyte™ S3 ZOOM cell imaging system and the fluorescence intensity was analyzed quantitatively using TissueQuest 6.0. (A) The fluorescence images showed that there was a huge increase of the fluorescence staining (green) in the CMs after treating the high dose of four compounds and aconitine, (B-C) the same as flow graphs and statistics, which indicated that high dose of S. tonkinensis induced apoptosis. The scale bar is 400 μm. Data are presented as the mean ± SEM, n ≥ 3. *P > 0.05, **P > 0.01, and ***P > 0.001 vs the control group. (TIF 23588 kb) [file 13287_2018_1126_MOESM1_ESM.tif]
